# Effects of Thymoquinone on Cell Proliferation, Oxidative Damage, and Toll-like Signaling Pathway Genes in H1650 Lung Adenocarcinoma Cell Line

**DOI:** 10.3390/medicina61101835

**Published:** 2025-10-14

**Authors:** Selen Karaoğlanoğlu, Gonca Gülbay

**Affiliations:** 1 Department of Pulmonology, School of Medicine, Ordu University, 52200 Ordu, Türkiye; 2 Department of Medical Biology, School of Medicine, Ordu University, 52200 Ordu, Türkiye

**Keywords:** cell proliferation, H1650 cell line, lung cancer, thymoquinone

## Abstract

*Background and Objectives*: Lung cancer is the leading cause of cancer-related mortality worldwide. In most cases, lung cancer is diagnosed at an advanced stage. For advanced-stage disease, treatment options are generally systemic and while novel treatment approaches offer hope, they may also lead to significant adverse effects. Therefore, alternative therapeutic strategies have been investigated for many years. Thymoquinone (TQ) is one such candidate. Previous studies have demonstrated its antioxidant, anti-inflammatory, antibacterial, and immunomodulatory properties. In our study, we aimed to evaluate the roles of TQ in the progression of H1650 lung adenocarcinoma cells. *Materials and Methods*: In this study, the antiproliferative effect of TQ on H1650 lung cancer cells was evaluated using MTT assay, its effect on oxidative damage was determined using 8-OHdG, and total antioxidant status (TAS), total oxidant status (TOS), and its effect on apoptosis were demonstrated using caspase-3 ELISA method. In addition, total RNA was extracted from both control and treatment groups, cDNA was synthesized, and mRNA expression changes of Toll-like receptor related genes (*TLR*) were analyzed using RT-PCR. *Results*: The decrease in the viability of H1650 lung cancer cells was observed in a time- and dose-dependent manner. The IC_50_ dose of TQ in the H1650 lung cancer cell line at 48 h was 26.59 µM. TQ treatment decreased the level of TOS and increased the level of TAS in H1650 lung cancer cells. Oxidative stress index decreased in the TQ-treated dose group in H1650 lung cancer cells. Elisa 8-OHdG and caspase-3 levels were not statistically significant. Compared to the control group, no statistically significant changes were observed in *TLR1*, *TLR2*, *TLR3*, *TLR4*, *TLR6*, *TLR7*, *TLR8*, and *TLR9* gene expressions in the treatment group treated with 26.59 µM TQ for 48 h. *Conclusions*: TQ shows potential as an anticancer agent and may contribute to the development of therapeutic approaches for lung cancers.

## 1. Introduction

Lung cancer remains the deadliest cancer worldwide, accounting for 1.8 million deaths [1]. It is the most commonly diagnosed cancer in men globally and ranks second in women after breast cancer [2]. In cases of non-small cell lung cancer (NSCLC) diagnosed at an early stage, surgical intervention is the primary treatment option. However, in advanced stages, treatment typically consists of systemic therapies such as chemotherapeutic agents and targeted therapies [3,4]. Most patients with lung cancer are diagnosed at an advanced stage, as the disease often does not cause noticeable symptoms in its early stages. Symptoms such as cough, chest pain, and fatigue generally appear after the tumor has grown and metastasized. Due to genetic instability, high tumor mutational burden, and immune evasion mechanisms, lung cancers tend to progress rapidly, grow aggressively, and metastasize early [5,6].

Lung cancer is a disease that develops not only due to genetic mutations but also due to the effects of chronic inflammation. Excessive or abnormal activation of *TLRs* can cause effects such as disruption of the immune system’s balance, maintenance of chronic inflammation in the tumor microenvironment, release of pro-tumor cytokines, attraction of immunosuppressive cells to the tumor site, and suppression of the anti-tumor immune response [7,8]. For example, various studies have shown that *TLR9* activation can enhance invasion and metastasis by increasing the expression of matrix metalloproteinases in tumor cells, while also supporting the angiogenesis process by increasing VEGF production. On the other hand, *TLR4* activation has been shown to increase the release of proinflammatory cytokines by activating signaling pathways such as MAPK. This will clearly lead to the continuation of chronic inflammation and support tumor cell proliferation [9,10].

Therefore, intensive systemic therapy is often required in the treatment of lung cancer. While these therapies are certainly life-saving, they also carry a significant risk of adverse effects [11,12,13,14]. Numerous studies have long been conducted on alternative therapeutic approaches to support conventional medicine and reduce treatment-related side effects [15,16,17]. The emergence of targeted therapies, particularly tyrosine kinase inhibitors, has revolutionized treatment strategies for specific subgroups of patients with genetic mutations such as alterations in the epidermal growth factor receptor (*EGFR*) gene [18]. However, the development of resistance mechanisms and the lack of durable responses highlight the need for continued exploration of alternative treatment strategies. Plant-derived bioactive compounds like Thymoquinone (TQ), curcumin, and resveratrol have demonstrated antiproliferative, proapoptotic, and antioxidant effects in various lung cancer cell lines and animal models [19,20,21].

TQ is the primary bioactive compound derived from *Nigella sativa* seeds and has been shown to inhibit proliferation, migration, and invasion in the A549 human lung adenocarcinoma cell line [22]. TQ has attracted attention as a potential natural agent that can enhance the efficacy of existing cancer therapies while reducing their associated side effects. The combined administration of TQ with other anticancer drugs has demonstrated synergistic effects, contributing to improved treatment outcomes. In various cancer types, TQ has exhibited a wide range of biological activities, including inhibition of cell proliferation, induction of apoptosis, suppression of invasion, metastasis, enhancement of chemosensitivity, anti-angiogenic effects, and anti-inflammatory properties [23]. In one study, TQ showed greater cytotoxic and apoptotic effects in laryngeal cancer cells without KRAS mutations compared to those with the mutation [24]. NSCLCs frequently harbor KRAS driver mutations, and half of these are KRAS. KRAS mutant NSCLC, mutated in conjunction with STK11 and/or KEAP1, is particularly resistant to conventional, targeted, and immunotherapy. KRAS is a gene that regulates cell division and growth. Monoallelic variants in KRAS cause uncontrolled cell division, leading to cancer development. Studies have shown that cancer cells with KRAS mutations are often resistant to certain chemotherapies and targeted therapies [25]. Furthermore, during the COVID-19 pandemic, TQ was reported to exert protective effects in cancer patients by reducing SARS-CoV-2 entry into host cells and mitigating multi organ damage [26].

In this in vitro study, we used the H1650 cell line, a well-characterized model of lung adenocarcinoma. The H1650 cell line is an NSCLC model in which the EGFR-PI3K/AKT pathways are active due to EGFR exon 19 deletion and PTEN loss. These characteristics create tumor microenvironment conditions that may affect the expression levels of genes belonging to the *TLR* family (*TLR1*, *TLR2*, *TLR3*, *TLR4*, *TLR6*, *TLR7*, *TLR8*, *TLR9*). Therefore, the H1650 cell line was chosen in our study to evaluate *TLR* gene expression profiles [27,28]. Our aim was to investigate the effects of TQ on cell proliferation, oxidative DNA damage, apoptosis, and the expression of Toll-like receptor signaling pathway genes in H1650 lung adenocarcinoma cells. To our knowledge, this is the first study to investigate the anticancer effects of TQ on the H1650 lung cancer cell line. Understanding the potential of TQ in lung cancer treatment may contribute to the development of novel therapeutic strategies and serve as a basis for future clinical research.

## 2. Materials and Methods

### 2.1. Cell Culture

TQ was purchased from Sigma-Aldrich (St. Louis, MO, USA). H1650 cancer cells (the H1650 cell line was obtained from Dr. Şakir Akgün, Kafkas University, Kars, Türkiye) were grown in RPMI 1640 (Gibco Laboratories, Grand Island, NY, USA) supplemented with 10% heat-inactivated fetal bovine serum (FBS, Ebsdorfergrund, Germany; Capricorn Scientific GmbH), 20 μg/mL streptomycin, 20 units/mL penicillin, 1 mM sodium pyruvate (Biological Industries, Beit-Haemek, Israel), and 0.1 mM amino acid solution (Biological Industries, Israel) and cultured at 37 °C in 5% CO_2_ in this study. We applied the active ingredient TQ to H1650 cells at different concentrations: 6.25 µM, 12.50 µM, 25.00 µM, 50.00 µM, 100.00 µM, and 200.00 µM.

### 2.2. Cell Viability Assay

The antiproliferative effects of TQ on H1650 lung cancer cells were determined using MTT [3-(4,5-Dimethylthiazol-2-yl)-2,5-Diphenyltetrazolium] assay at a concentration of 2.10^4^ cells per well in 96-well plates according to the kit’s instructions (Cell Proliferation Kit; GoldBio, St. Louis, MO, USA). After the dose intervals were completed, the MTT combination was applied in accordance with the dose and duration recommended by the manufacturer and measured spectrophotometrically. The protocols of the relevant kits were followed. Absorbance (570 nm) was measured using the Biotek Epoch 2 microplate reader and Gen5 software (Gen5 version 3.10).

Cell Viability (%) = [(Treatment group OD_570_ − Blank well OD_570_)/(Untreated group OD_570_ − Blank well OD_570_)] × 100%

The IC_50_ dosage was used as the dose group in the other molecular studies of this study.

#### 2.2.1. Total Antioxidant Status and Total Oxidant Status Assay

The Rel Assay TAS and TOS kit (Rel Assay Kit Diagnostics, Gaziantep, Turkey) was used to determine the effects of TQ on the total antioxidant and oxidant status in H1650 lung cancer cells. The study was conducted in accordance with the manufacturer’s instructions. TAS and TOS values were measured using the Biotek Epoch 2 (Santa Clara, CA, USA) microplate reader and Gen5 software (Gen5 version 3.10). TAS and TOS value was calculated according to the following formula.

TOS = [(ΔAbsSample)/(ΔAbsStandard) × Concantration of standard]

TAS = [(ΔAbs H_2_O) − (ΔAbs Sample)/(ΔAbs H_2_O) − (ΔAbs Standard)]

#### 2.2.2. Oxidative Stress Index Assay

The Oxidative Stress Index (OSI) serves as a measure of oxidative stress levels and is a unitless parameter calculated by taking the ratio of TOS to TAS [29]. To facilitate this calculation, TAS was initially converted from mmol Trolox equivalents per liter to µmol Trolox equivalents per liter.

OSI = [(TOS, µmol H_2_O_2_ equivalents/L)/(TAS, µmol Trolox equivalents/L) × 100].

#### 2.2.3. 8-Hydroxy-2′-Deoxyguanosine Assay

Human 8-Hydroxy-2 Deoxyguanosine (8-OHdG) biomarker, which indicates DNA damage caused by oxidative stress, was determined by ELISA method. 8-OHdG (Bioassay Technology Laboratory, BT Lab, Shanghai, China) determination was carried out by following the BT Lab (Shanghai, China) kit and protocols. The detection range was 50 to 800 ng/mLfor 8-OHdG.

#### 2.2.4. Caspase-3 Assay

The concentration of protein caspase-3 in TQ H1650 lung cancer cells was evaluated using the ELISA Kit from BT Lab (Bioassay Technology Laboratory, BT Lab, Shanghai, China) according to the protocols provided by the manufacturer. The detection range was 0.75 to 12 ng/mL for Caspase-3.

#### 2.2.5. Total RNA Isolation, cDNA Synthesis, and RT-PCR Analysis

Total RNA isolation from cells was carried out using trizol (Invitrogen, Life Technologies, Carlsbad, CA, USA) in accordance with the manufacturer’s instructions. The A.B.T. synthesis kit with RNase Inhibitor was used for cDNA synthesis (A.B.T, Laboratory Industries, Ankara, Türkiye). Real-time PCR (RT-PCR, Rotor-Gene Q, Hilden, Germany) was used to assess changes in mRNA expression of (Toll-like receptor genes, *TLR*) *TLR1*, *TLR2*, *TLR3*, *TLR4*, *TLR6*, *TLR7*, *TLR8*, and *TLR9*. Normalization was accomplished through the use of Beta-Actin (ACTB). The primer sequences used in this study were given in Table 1 [30]. RT-PCR was used to perform real-time PCR (Rotor Gene Q, Qiagen) tests using the SYBR Green qPCR Master Mix ABTTM 2X qPCR SYBR-Green Master Mix (Turkey) protocol.

### 2.3. Statistical Analysis

All statistical analyses were carried out using GraphPad Prism 9.4.1 software (GraphPad Software, San Diego, CA, USA). The real-time PCR quantification was performed via the 2^−∆∆CT^ method, with further analysis using the Gene Globe RT-PCR Analysis RT2 Profile PCR Array Data Analysis platform (Qiagen). Values were reported as mean ± S.D. For the comparison of multiple groups, we used analysis of variance (one-way ANOVA) to assess statistical significance between the groups. Post-hoc comparisons were performed using Dunnett’s test, to compare each mean with a control mean. The normality of data distribution was assessed using the Shapiro–Wilk test to determine the suitability of parametric or non-parametric statistical tests. When comparing two groups, either an unpaired *t*-test or Mann–Whitney test was used, depending on the distribution of the data. A *p*-value of <0.05 was considered statistically significant (ns *p* > 0.05, * *p* ≤ 0.05).

## 3. Results

### 3.1. MTT Analysis

**Figure 1:**
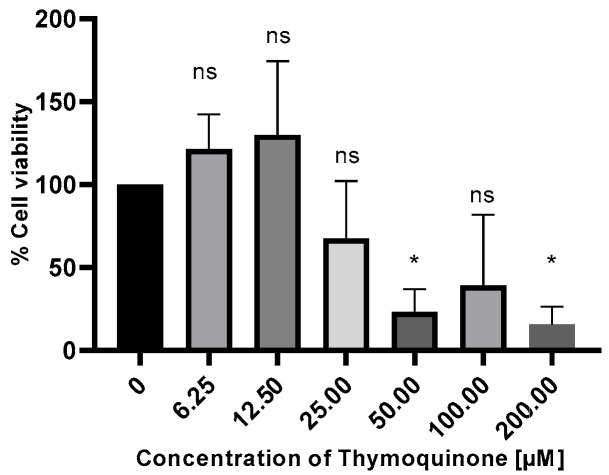
H1650 lung cells were treated with TQ at different concentrations and time intervals, and their viability was assessed by MTT assay, ns, non-significant *p* > 0.05; * *p* ≤ 0.05.

Upon treatment with TQ, the viability of H1650 cells was assessed using the MTT assay. A decrease in cell viability was observed in a time- and dose-dependent manner. The half-maximal inhibitory concentration (IC_50_) dose of TQ was determined as 26.59 µM at 48 h in the H1650 cell line (Figure 1).

### 3.2. Total Antioxidant Status (TAS), Total Oxidant Status (TOS), and Oxidative Stress Index (OSI) Analysis

**Figure 2:**
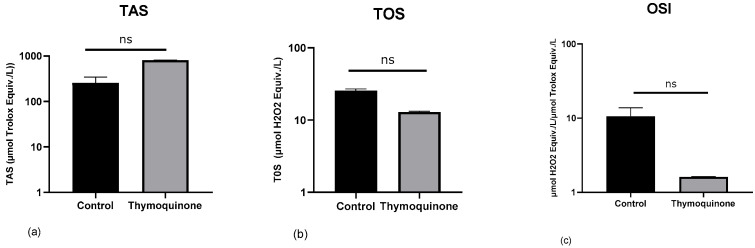
Data are presented as mean ± SD. The *Y*-axis is plotted on a log10 scale. Modulation of the TAS, TOS, and OSI values in H1650 lung cancer cells by TQ. (**a**) TAS (control Group: 254.2 ± 89.89, TQ-treated: 803.0 ± 8.98), (**b**) TOS (control Group: 25.57 ± 1.432, TQ-treated: 12.91 ± 0.358), (**c**) OSI (control Group: 10.62 ± 3.191, TQ-treated: 1.60 ± 0.026).

To determine the effects of TQ on oxidative stress in H1650 lung cancer cells, TAS, TOS, and OSI values were calculated. TQ treatment decreased the total oxidant status level and increased the total antioxidant status level in H1650 lung cancer cells (TAS, control group: 254.2 ± 89.89, TQ-treated: 803.0 ± 8.98, *p* = 0.33; TOS, control group: mean 25.57 ± 1.432, TQ-treated: mean 12.91 ± 0.358, *p* = 0.33) (Figure 2a,b). Oxidative stress index decreased in the TQ-treated dose group in H1650 lung cancer cells (control group: mean 10.62 ± 3.191, TQ-treated: mean 1.60 ± 0.026, *p* = 0.33) (Figure 2c). The administration of TQ appears to suppress the generation of reactive oxygen species (ROS), thereby mitigating oxidative stress. Elevated ROS levels are recognized as key modulators in the activation of redox-sensitive signaling cascades, which contribute significantly to oncogenesis and tumor progression.

### 3.3. 8-Hydroxy-2′-Deoxyguanosine (8-OHdG) Analysis

**Figure 3:**
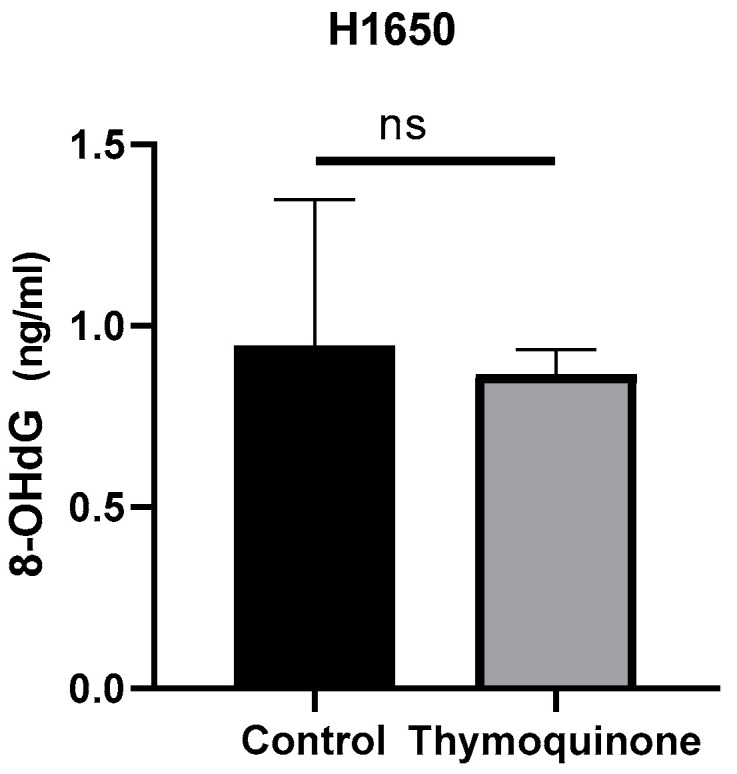
8-OHdG concentration levels of TQ-treated H1650 lung cancer cells and controls (ns, non-spesific; control group: 0.94 ± 0.401 ng/mL, TQ-treated: 0.86 ± 0.069 ng/mL, *p* > 0.99).

8-OHdG level was investigated by ELISA method. Changes in protein concentration were determined by comparing TQ-treated H1650 lung cancer cells with the control group. According to the data, the mean 8-OHdG concentration in the H1650 control group was 0.94 ± 0.401 ng/mL, while it was 0.86 ± 0.069 ng/mL in TQ-treated cells. When the results were compared statistically, no significant difference was found (*p* > 0.99, Figure 3).

### 3.4. Caspase-3 Analysis

**Figure 4:**
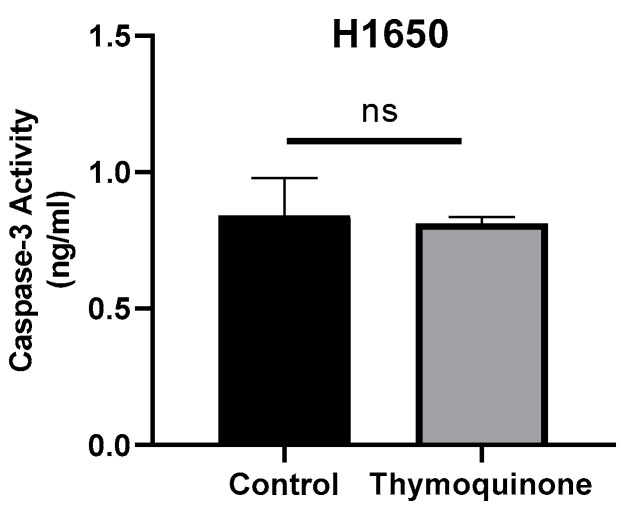
Caspase-3 concentration levels of TQ-treated H1650 lung cancer cells and controls (ns, non-spesific; control group: 0.84 ± 0.137, TQ-treated 0.81 ± 0.023 *p* > 0.99).

To determine whether TQ further induced caspase-3 activation in H1650 lung cancer cells, caspase-3 protein levels were examined using ELISA. Accordingly, the mean caspase-3 levels in cells were determined as 0.84 ± 0.137 for the control group and 0.81 ± 0.023 for TQ-treated H1650 cells. No significant difference was found when the results were statistically compared (*p* > 0.99, Figure 4).

### 3.5. Real Time-PCR Analysis

After total RNA was isolated from control and dose group cells, the cDNA synthesis was performed. The expression analysis of *TLR1*, *TLR2*, *TLR3*, *TLR4*, *TLR6*, *TLR7*, *TLR8,* and *TLR9* was performed by RT-PCR according to the SYBR Green qPCR master mix protocol. As a result of RT-PCR, mRNA expression changes of genes involved in the apoptosis pathway were evaluated.

In the group treated with 26.59 µM TQ at the 48th hour, no significant changes were observed in the gene expressions of *TLR1* (1.24-fold, *p* = 0.28), *TLR2* (1.25-fold, *p* = 0.45), *TLR3* (1.38-fold, *p* = 0.42), *TLR4* (1.87-fold, *p* = 0.06), *TLR6* (1.18-fold, *p* = 0.30), *TLR7* (1.62-fold, *p* = 0.09), *TLR8* (1.99-fold, *p* = 0.06), and *TLR9* (1.27-fold, *p* = 0.33) when compared to the control group.

## 4. Discussion

Lung cancer remains one of the leading causes of cancer-related mortality worldwide, with NSCLC accounting for approximately 85% of all cases. Despite advances in diagnosis and treatment, the prognosis for lung cancer patients remains poor, underscoring the urgent need for novel therapeutic strategies. In recent years, increasing attention has been directed toward naturally derived compounds with potential anticancer properties. TQ, the bioactive component of *Nigella sativa*, has shown promising effects in various cancer models due to its pro-apoptotic, antioxidant, and immunomodulatory activities. This is the first study to evaluate the effects of TQ on cell viability, oxidative stress parameters, apoptosis markers, and *TLR* gene expression in the H1650 NSCLC cell line, investigating its anticancer potential. Our findings contribute to the growing body of evidence supporting the therapeutic potential of TQ in lung cancer.

Karimian et al. [15] investigated the anticancer effects of TQ on the A549 lung cancer cell line and reported an IC_50_ value of 40 µM. Khan et al. [31] examined the anticancer effects of TQ at 24, 48, and 72 h across various small cell lung cancer cell lines. According to their findings, H446 cells were the most sensitive to TQ at different incubation times, while DM579 cells exhibited the greatest resistance, particularly after 24 h of exposure. The same study also demonstrated the tumor-suppressive effects of TQ in vivo. Similarly, Samarghandian et al. [32] evaluated the effects of TQ in a dose- (25, 50, and 100 µM) and time-dependent (24, 48, and 72 h) manner on A549 and MRC5 lung cancer cell lines. Their results showed that TQ significantly inhibited the viability of A549 cancer cells in both a dose- and time-dependent manner, reducing cell viability by more than 60%. However, this effect was not observed in the noncancerous MRC5 cell line. Gurbilek et al. [33] calculated the IC_50_ value of TQ at 113.49 µM after 24 h in an NSCLC cell line.

The antiproliferative effects of TQ have also been investigated in various cancer types other than lung cancer. Osorio-Perez et al., reported IC_50_ values of 82.59 µM and 55.83 µM for TQ in colon (HCT 15) and prostate (PC3) cancer cell lines, respectively [34]. Seçme et al. [35] demonstrated the antiproliferative effect of TQ in a breast cancer cell line (MCF-7) with an IC_50_ value of 7.867 µM. He et al. [36] investigated the effect of TQ on cell viability in gastric cancer cell lines HGC-27 and MKN-45 and showed that increasing TQ concentrations inhibited cell viability more effectively using CCK-8 (cell counting kit-8) assay. In our study, we determined the IC_50_ value of TQ in H1650 lung cancer cell line as 26.59 µM after 48 h.

Oxidative stress (OS), which occurs when ROS production exceeds the body’s antioxidant defense mechanisms, plays a complex and important role in cancer. High levels of ROS can cause oxidative damage, triggering gene mutations and activation of oncogenic signaling pathways that are hallmarks of cancer initiation and progression [37]. Oxidative stress not only directly contributes to DNA damage and tumor progression but also acts as a key driver of chronic inflammation, which further strengthens the tumor-promoting microenvironment. Chronic inflammation is a fundamental contributor to cancer development and progression by promoting tumor initiation and metastasis through the production of pro-inflammatory cytokines, reactive oxygen species, and growth factors. Inflammatory microenvironments can promote genetic mutations, inhibit apoptosis. and facilitate angiogenesis, all of which are critical in tumor formation [38].

The effects of TQ on inflammation and oxidative stress are well documented [39,40]. Shakeel et al. [41] showed that TQ significantly increased mitochondrial ROS production in H1299 and A549 lung cancer cell lines. Khan et al. [39] reported that the anti-inflammatory activity of TQ was mediated by inhibition of the inducible nitric oxide synthase (iNOS) pathway and reduction of cyclooxygenase-2 (COX-2) expression. In our study, treatment with TQ at a concentration of 26.59 µM IC_50_ resulted in an increase in TAS (dose group: 803.0, control group: 254.2) and a decrease in TOS (dose group: 12.91, control group: 25.57). However, although the decrease in OSI in the group treated with TQ (dose group: 1.60, control group: 10.62) was significant, it was not reflected in the statistical results. We believe that this situation may have been due to intra-group variations preventing the results from reaching the significance threshold.

The oxidative form of DNA damage induced by oxidative stress is 8-OHdG. Karimian et al. [15] reported an increase in 8-OHdG levels in TQ-treated A549 lung cancer cells compared to the control group. In contrast to this study, in our study we measured 8-OHdG levels, a marker of oxidative DNA damage, at 0.94 ng/mL and 0.86 ng/mL in the control dose groups, respectively. Although our results were not statistically significant, they may indicate that the H1650 cell line is more sensitive to the DNA-damaging effects of TQ. The culture duration used in this study was sufficient for the evaluation of many cytotoxic and oxidative stress parameters; however, longer culture protocols may be required to more clearly demonstrate DNA damage markers such as 8-OHdG. This should be taken into consideration in future research.

This natural compound activates apoptosis and has also been shown to activate tumor-suppressing genes in different cell lines. In two separate studies using human colon cancer (HCT116) and breast cancer cells (MCF7), it has been shown to induce apoptosis via the P53 mechanism [42,43].

Alam et al. [44] showed that TQ induced the pro-apoptotic protein, Bax, while inhibiting the anti-apoptotic protein Bcl-2 in H1299 and A549 NSCLC cells. In the same study, it was also emphasized that the combined use of TQ and Quercetin gave more effective results. Zhu et al. [45] reported that TQ inhibited pulmonary arterial remodeling through the P38MAPK/NF-κB signaling pathway and showed a partially therapeutic effect on induced pulmonary arterial hypertension in rats. In the study by Samarghandian and et al. [32], TQ dose dependently activated both caspase-3 and caspase-9 in lung cancer cells compared to control cells. Dera et al. [46] demonstrated the pro-apoptotic effect of TQ in combination therapy with indirubin-3-monoxime both in vivo and in vitro by annexin assay, along with a decrease in the Bcl-2/Bax ratio. Caspase-3 is the main executor of proteolytic degradation in cells exposed to cytotoxic drugs, radiotherapy, or immunotherapy.

In our study, we analyzed caspase-3 protein levels using the ELISA method to determine whether TQ further induced caspase-3 activation in H1650 lung cancer cells. Accordingly, we determined the mean serum caspase-3 levels as 0.84 ± 0.137 in the control group and 0.81 ± 0.023 in TQ-treated H1650 cells. The results revealed that there was no statistically significant difference between the groups.

In humans, *TLRs* are components of the innate immune system [30]. Altered expression of *TLRs* has been reported in several cancer types, including prostate [47], liver [48], gallbladder [49], and endometrial cancer [50]. In the study conducted by Pomerenke et al., increased TLR activation was observed in patients with COPD, leading to increased TNF-α secretion [51]. However, studies investigating the effect of TQ on *TLR* expression in lung cancer types are extremely limited. In our study, although we observed an increase in *TLR1*, *TLR2*, *TLR3*, *TLR4*, *TLR6*, *TLR7*, *TLR8,* and *TLR9* expression levels in the H1650 lung cancer cell line following TQ treatment, the results were not statistically significant.

This study has certain limitations. Firstly, the experimental findings were obtained using only a single cell line. This may constitute a certain limitation in terms of the generalizability of the results. However, the cell line used is a well-characterized model in [relevant biological process/disease] research, and the data obtained provide important preliminary findings for the literature. In future studies, validating these results in different cell lines would be beneficial in further strengthening their biological validity.

## 5. Conclusions

This study contributes to the growing body of evidence demonstrating the anticancer potential of TQ in lung cancer. In our experimental model using the H1650 NSCLC cell line, TQ exhibited a notable cytotoxic effect with an IC_50_ value of 26.59 µM at 48 h. Furthermore, TQ was shown to modulate oxidative stress by increasing TAS and decreasing TOS and OSI. Although 8-OHdG levels, an indicator of oxidative DNA damage, did not significantly differ between the treatment and control groups, the trend was consistent with existing literature on TQ’s pro-oxidant effects in cancer cells. Caspase-3 activation, a hallmark of apoptosis, was evaluated but did not show a statistically significant increase, suggesting that TQ-induced apoptosis in H1650 cells may be mediated through caspase-independent or alternative pathways. While upregulation of *TLR* gene expression was observed, the results were not statistically significant, highlighting the need for further investigation into the immunomodulatory role of TQ. Taken together, our findings support the potential of TQ as an anticancer agent and warrant further studies to elucidate its molecular mechanisms in NSCLC and its interaction with inflammatory and apoptotic pathways.

## Figures and Tables

**Table 1:** Real-time PCR forward and reverse prime sequences [30].

Gene Names	Forward	Reverse	Amplicon Length (bp)
	(5′–3′)	(5′–3′)	
*TLR1*	CAGCGATGTGTTCGGTTTTCCG	GATGGGCAAAGCATGTGGACCA	1151
*TLR2*	TTATCCAGCACACGAATACACAG	AGGCATCTGGTAGAGTCATCAA	160
*TLR3*	GGCTAGCAGTCATCCAACAGAA	GCAGTCAGCAACTTCATGGC	143
*TLR4*	CCCTGAGGCATTTAGGCAGCTA	GGTAGAGAGGTGGCTTAGGCT	144
*TLR6*	TTCTCCGACGGAAATGAATTTGC	CAGCGGTAGGTCTTTTGGAAC	75
*TLR7*	CTTTGGACCTCAGCCACAACCA	CGCAACTGGAAGGCATCTTGTAG	163
*TLR8*	ACTCCAGCAGTTTCCTCGTCTC	AAAGCCAGAGGGTAGGTGGGAA	144
*TLR9*	TGAGCCACAACTGCATCTCGCA	CAGTCGTGGTAGCTCCGTGAAT	116
*Beta–Actin*	CACCATTGGCAATGAGCGGTTC	AGGTCTTTGCGGATGTCCACGT	131

## Data Availability

The data sets and analyses from the study can be obtained from the corresponding author upon reasonable request.

## Abbreviations

The following abbreviations are used in this manuscript:

NSCLC	Non-small cell lung cancer
TQ	Thymoquinone
TLR	Toll-like receptor
EGFR	Epidermal growth factor receptor
TAS	Total antioxidant status
TOS	Total oxidant status
OSI	Oxidative stress index
8-OHdG	Human 8-Hydroxy-2 Deoxyguanosine
RT-PCR	Real-time PCR
ROS	Reactive oxygen species
OS	Oxidative stress
iNOS	Inducible nitric oxide synthase
COX-2	Cyclooxygenase-2
